# Regression of Urrets-Zavalia Syndrome After Deep Lamellar Keratoplasty for Keratoconus: A Case Study

**DOI:** 10.2174/1874364100802010130

**Published:** 2008-08-08

**Authors:** Leopoldo Spadea, Mariangela Viola, Giovanna Viola

**Affiliations:** Eye Clinic, San Salvatore Hospital, Surgical Science Department, University of L'Aquila, L’Aquila, Italy

**Keywords:** Dapiprazole, DLK, keratoconus, pilocarpine, Urrets-Zavalia syndrome.

## Abstract

We report a case of pharmacologic regression of Urrets-Zavalia syndrome, following deep lamellar keratoplasty for severe keratoconus. Sympatholytic and parasympathomimetic drops were administered and a progressive reduction of mydriasis and restoring of pupillary kinetics were observed. Four years after surgery, it was resulting in only slight residual anisocoria.

## INTRODUCTION

Urrets-Zavalia syndrome (UZS) was first identified as an uncommon and irreversible complication after penetrating keratoplasty for keratoconus, in a series of 6 cases with fixed, dilated pupil, iris atrophy and secondary glaucoma [1]. It was also described after deep lamellar keratoplasty (DLK) and uncomplicated trabeculectomy [2, 3]. We report a case of microperforation of the Descemet membrane during DLK for a severe keratoconus and development of fixed dilated pupil syndrome, after air injection into the anterior chamber.

## CASE REPORT

**Fig. (1):**
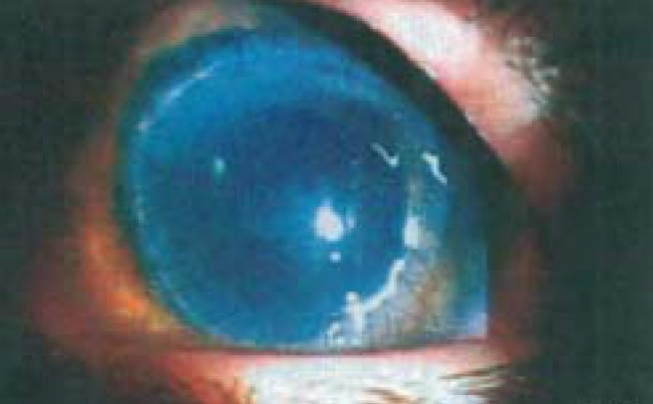
Two days after DLK. The pupil was fixed of 9 mm. The cornea was edematous, with a 24-bite 10/0 nylon single running suture.

**Fig. (2):**
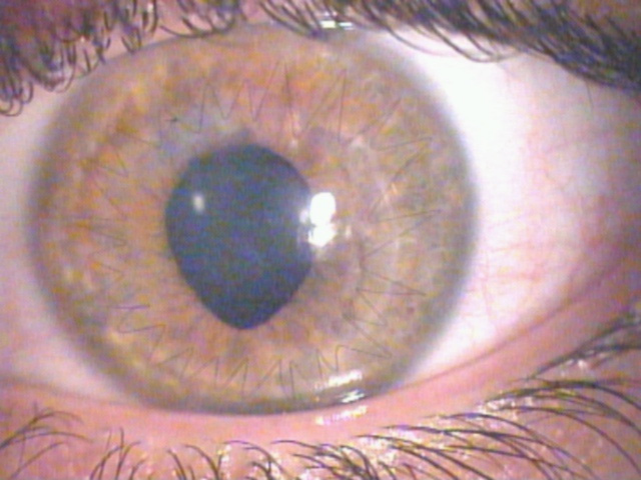
Three months after surgery. The horizontal and the vertical pupil diameter were, respectively, 4 mm and 6 mm with a normal kinetics. The cornea was clear with a 24-bite 10/0 nylon single running suture.

**Fig. (3):**
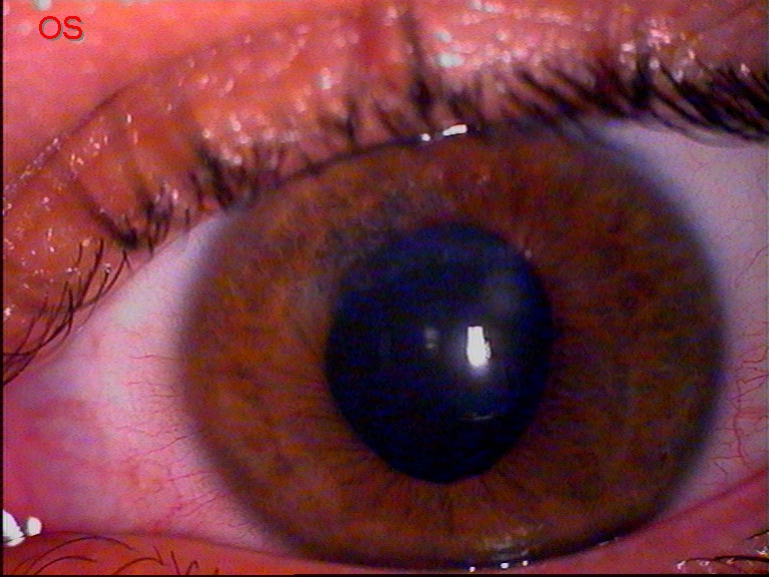
Four years after surgery. The horizontal and the vertical pupil diameter were, respectively, 4 mm and 4.5 mm with a normal kinetics. A small supero-nasal zone of iris atrophy is present. The graft remained very clear.

A 24 years old man, affected by bilateral keratoconus, was submitted to DLK in the left eye. The preoperative uncorrected visual acuity (UCVA) was 20/600 and the best corrected visual acuity (BCVA) was 20/63; the pachymetry was 378 μm. After peribulbar anaesthesia (Chloride Mepivacaine 2%) and 8 mm / 300 µm trephination with Hanna trephine (Moria Surgical, Antony, France), using a disk knife, a slice of superficial cornea was removed. Therefore a phototherapeutic keratectomy (PTK) of 20 µm, using MEL 70 excimer laser (Carl Zeiss Meditec, Jena, Germany) was performed to improve the smoothness of the corneal bed. During the final phase of photoablation, a microperforation of Descemet membrane was noted, so the photoablation was stopped and air was introduced in the anterior chamber. Then an 8.5 mm donor cornea without Descemet and endothelial layers was placed to the host bed with a 24-bite nylon single running suture. Finally, an air bubble was left in the anterior chamber to reduce the risk of aqueous seepage between the posterior corneal lamella and the lamellar graft. On the first postoperative day, the graft was edematous and an aqueous seepage between the corneal graft and the host bed (a doubled anterior chamber) was observed. Therefore, a further air bubble was injected and left in the anterior chamber to appose the host donor lamellar graft interface. The following day, the cornea was less edematous with a big air bubble in the anterior chamber. No double anterior chamber was observed. No mydriatic drops were used, but the pupil was fixed and dilated (9 mm) (Fig. **1**). The intraocular pressure (IOP) was 30 mmHg. After a complete corneal re-epithelialization (7 day), topical corticosteroid (butyrate clobetasone 0.1%) drops were administered for 1 month, then tapered in 15 days. In the following two weeks, a clear cornea was observed, but the pupil remained fixed, dilated and no responsive to light stimulus. The IOP was 24 mmHg and timolol 0.50% drops (2 times a day) was given. Three weeks after the surgery, the IOP was 12 mmHg and the pupil diameter was still 8 mm, as described in UZS. Therefore, sympatholytic (dapiprazole 0.5%) drops four times a day and parasympathomimetic (pilocarpine 2%) drops three times a day were prescribed. No peripheral iridectomy or other surgical procedures were performed. One week later, the pupil reduced to 6 mm and the therapy was progressively tapered. Three months after surgery, the cornea was clear, the UCVA was 20/200, the BCVA was 20/40, and the IOP was 12 mmHg. The horizontal pupil diameter was 4 mm with a normal kinetics and a quite oval pattern (Fig. **2**). At six months, the suture was removed. Four years later, the pupil shape was normal with only a slight residual anisocoria (Fig. **3**). The UCVA was 20/200, the BCVA was 20/20, the keratometric astigmatism was 1.8 D (ax 75°) and the IOP was 14 mmHg without any therapy.

## DISCUSSION

The etiology of UZS after keratoplasty is not clear up till now. Many Authors discussed about the possible causal factors: strong mydriasis that brought on peripheral anterior synechiae and glaucoma [1], iris ischemia developed after iris compression between the lens and the cornea during surgery [4], an abnormal immunological, neurological and iris in keratoconic eyes [5], IOP rise [6], direct iris trauma during surgery [2, 3], preexisting anterior synechiae [7]. In 2002, some authors have reported cases of UZS developed after DLK with intraoperative microperforation of Descemet membrane and air injection in the anterior chamber. They suggested that the air bubble in the anterior chamber may cause a pupil block, raised intraocular pressure and secondary iris ischemia with a dilated, fixed pupil [2, 8]. Gasset *etal*. observed that in keratoconic eyes the pupils remain dilated for longer periods after mydriasis than in normal eyes. They suggested a strong reaction to the application of mydriatics as far as speed of dilation and duration of effect is concerned; this observation is also seen in the eyes of patients with Down’s syndrome [5]. An abnormality of the sympathetic nervous system in the keratoconic eye, remains unproven. Tuft and Buckley suggested in the presence of raised IOP, the low ocular rigidity of the keratoconic eye permits occlusion of the vessels at the root of iris within the sclera, which results in iris ischemia, while preserving ciliary body function [6].

In 1983 Lagoutte *et al*. described an early resolution of UZS with an association of sympatholytic and parasympathomimetic drops and they suggested a physiophatological concept of sympathetic spasm with parasympathetic inhibition [9]*. *In our case, the compressive and ischemic theory may play a role, because iris ischemia could develop after raised intraocular pressure and leave a fixed dilated pupil, however, the late resolution of the fixed pupil with drugs lend to support the theory of intrinsic iris neurological abnormalities in keratoconus.

In conclusion, the presented case is the first to our knowledge with such a late resolution of UZS (21 days after surgery) by using a pharmacological therapy with sympatholytic and parasympathomimetic drops. This report could indicate a reversible mechanism involved in the UZS that may be modulated by the additive effect of dapiprazole and pilocarpine for a longer time.
